# Evaluation of Patient Satisfaction With the New Web-Based Medical Appointment Systems “Mawid” at Primary Health Care Level in Southwest Saudi Arabia: A Cross-Sectional Study

**DOI:** 10.7759/cureus.34038

**Published:** 2023-01-21

**Authors:** Mohammed Salih Mahfouz, Majed A Ryani, Abdulrahem A Shubair, Saud Y Somili, Ali A Majrashi, Hussam Abdu Zalah, Adel Ali Khubrani, Mohammed I Dabsh, Abdullatif M Maashi

**Affiliations:** 1 Department of Family and Community Medicine, Jazan University, Jazan, SAU; 2 Faculty of Medicine, Jazan University, Jazan, SAU

**Keywords:** “mawid”, jazan region, patients satisfaction, appointment system, web-based

## Abstract

Background

Patient satisfaction has become an influential corner in the health services process. Web-based appointment scheduling has been expanded for its benefits and has become a popular research topic. This study’s objectives were to assess patients’ satisfaction and perception with the new Web-Based Medical Appointment System “Mawid” program and determine the associated factors at the Primary Health Care Centers level in Jazan Southwest Saudi Arabia.

Methods

An observational cross-sectional survey was implemented among 424 adults aged 18 years and above, attending a randomly selected 12 primary health care centers in the Jizan region, Southwest Saudi Arabia. The study instrument included socio-demographic background information, perception, and level of satisfaction with the new appointment system. Responses were analyzed using the SPSS program by applying descriptive and inferential statistical techniques.

Results

The overall level of satisfaction was very high at 94.3% with 95% C.I. (91.7-96.1). A large proportion of study participants were highly satisfied with the new Web-Based Medical appointment System “Mawid” as nine satisfaction items scored a level of satisfaction of 90% and above. Regarding the perception, 89.1% of the participants agreed that the appointment booking system regulates the number of patients, while 87.7% of participants considered that the appointment system reduces clinic crowding. More than half of respondents (61.8%) agreed that the community culture might limit the scheduling system’s use. Univariate and multivariate logistic regression analysis suggested that male patients were more likely to have a higher level of satisfaction as compared with female (COR= 2.95, 95% C.I.:1.15-7.60, *p *= 0.025) and (AOR= 3.12, 95% C.I:1.14-8.52, *p *= 0.026), respectively.

Conclusions

In conclusion, this study revealed a high level of satisfaction among study the participants with the new Web-Based Medical Appointment System “Mawid.” The system effectively improved patients’ satisfaction with registration and reduced waiting times. Patients' satisfaction can be assessed regularly and used systematically as a quality and benchmarking instrument in primary health care.

## Introduction

Patient satisfaction has become an influential corner in health system planning and development. It is a crucial goal of healthcare organizations and presents challenges to providing quality patient care [1]. Saudi Ministry of Health is increasingly insisting on the role of patient satisfaction in the care process, manifested by its Patient Experience Measurement Program [2].

Nowadays, because of the expanded challenge in providing excellent healthcare services benefits by suppliers, patient fulfillment has become one of the needs of well-being focuses [3]. When the appointment scheduling system is planned and actualized dependent on the patient's needs and wishes, it enables suppliers to more readily comprehend the patient's issues, recognize the framework shortcomings, increment understanding fulfillment, and improve clinical outcomes [4].

The Saudi Ministry of Health (MOH) launched many health initiatives related to the National Transformation Program (NTP) 2020 and the Saudi Vision 2030, among them e-health, which seeks to provide health services that are needed, at a convenient location, via web-based services. The new “Mawid” system, is an electronic service that enables patients or users to book, cancel or reschedule their appointment at primary healthcare centers (PHCCs). The system available in smartphone applications facilitates the electronic scheduling of appointments in PHCCs. Further, the application books the appointment and confirms or cancels it in any hospital referred to the patient through convenient channels the application provides to its users [5].

The advantages of these applications are that it shortens the patient “waiting” time in front of doctors' clinics. It also makes the doctor aware of what he is doing for that day in terms of readiness [6]. Overall, the literature suggests a growing trend for the adopting web-based appointment systems to save time and resources [7-12].

Several studies assessing web-based systems documented their efficiency, and most patients would use the service again [7-17]. To date, there are few studies about the efficacy of web-based appointment systems in Saudi Arabia [18]. Hence, this study’s objectives were to assess patients’ satisfaction and perception with the new web-based medical appointment system “Mawid” program and determine the associated factors at the PHCCs level in Jazan Southwest Saudi Arabia.

## Materials and methods

Study design, place, and participants

An analytical cross-sectional study was conducted to evaluate patients’ perception and satisfaction with the web-based medical appointment systems “Mawid” in PHCCs in Jazan Region. The study was conducted in the Jazan region, located in the southwest of the Kingdom of Saudi Arabia. The region includes a number of governorates and administrative centers and is distributed into six health sectors: central, Middle, Southern, Northern, Eastern, and Western. The region is highly populated, and the estimated population is about 1,567,547 people, according to statistics in 2017 [19]. The study included all adult patients, age 18 and above who attended the selected PHCCs during the study period and agreed to sign the study consent; we excluded participants who could not speak Arabic.

Sample size and sample design

Jazan region administratively comprises six health sectors; the sampling design was multi-stage stratified random sampling. In the first stage, two PHCCs were selected from the six health sectors of the Jazan region. In the second stage, patients were selected randomly from each selected PHCCs using systematic random sampling. This study's sample size was calculated using the sample size formula for cross-sectional study design. The formula is developed to calculate a representative sample for proportions and written as follows: (n= Z^2^_(1−α/2) _×P(1−P)/d^2^). The sample's anticipated population proportion (p) is set to be 50% because this is the safest choice for (p) since the sample size required is largest when P=50%. For a 95% confidence level, Z^2^_(1−α/2)_ = 1.96, error (d), not more than 5%, then the initial sample size will be 400, and finally, after accounting for a 10% non-response rate, the required sample size increased to 440 persons.

Data collection method and study instrument

The data were collected by interviewing patients face to face to fill out the study questionnaire. A structured paper-based questionnaire was used for data collection. The tool utilized for this current research aimed to elicit information on evaluating patients’ experience attending PHCCs in the Jazan region regarding their satisfaction and perception of the Medical Appointment System “Mawid” among the target population, is designed after consulting the relevant literature [7-12]. The questionnaire is divided into four sections: The first section contains demographic data and medical history, such as age, gender, occupation, level of education, marital status, and residence. The second section contains the participants’ Health information and chronic conditions like diabetes or hypertension and the answer to these questions are (yes or no). The third section involves information on the level of satisfaction with the service. The questions in this section are Likert-based questions answered (Strongly Satisfied, Satisfied, Uncertain, Dissatisfied, Dissatisfied Strongly). Finally, the fourth section involves questions on patients’ perception of the Medical Appointment System “Mawid.” The questions in this section are also based on Likert scale and answered (I strongly agree, agree, disagree, disagree or disagree strongly).

Piloting, reliability, and validity of the instrument

The main objective of the pre-testing was to test the adequacy and content of the questionnaire, sample design, and fieldwork plan. The length of the interview, identification of the respondents, perception of the respondents towards the contents of the questionnaire, and sequences of topics are tested through pre-testing operation. Pre-testing of the questionnaire is conducted in two PHCCs, through the distribution of 30 questionnaires. The pilot study also was used to assess the reliability. The study questionnaire provided a Reliability Statistics Cronbach’s Alpha of Items 0.74126. Experts assessed the face validity of the questionnaire with a deep understanding in the family and community medicine department.

Data management and statistical analysis 

The completed questionnaires were reviewed on daily base to avoid mistakes. The SPSS (Statistical Package for Social Sciences) software program was used for data analysis. The results of this research are presented using frequency tables, percentages, means, SD, 95% CI and crude and adjusted odds ratios. For data analysis, the lowest score was valued as (1) for strongly dissatisfied and the highest as (5) for strongly satisfied. The overall level of satisfaction was calculated from 10 statements on the satisfaction on the web-based and dichotomized into two satisfied and dissatisfied. Chi-squared test for independence and the independent t-test was also used to determine the differences between male and female response. Moreover, bivariate and multivariable logistic regression analyses were used to determine the association between background characteristics and the overall level of satisfaction. A *p*-value less than 0.05 was used to indicate the statistical significance.

Ethical consideration 

This study was conducted in accordance with the ethical standards of the Kingdom of Saudi Arabia and the Helsinki Declaration. All the participants, read, understood and signed a written consent form. Participants were told that they have the freedom to participate or to withdraw from the study at any time. The anonymity of participants was emphasized, and confidentiality was strictly maintained on all collected questionnaires. Also, approval was obtained from the Standing Committee for Scientific Research Ethics - Jazan University (HAPO-10-Z-001) reference (REC40/3-080).

## Results

Overall, 421 (of 440) attendees to PHCCs in the Jazan region completed the questionnaires giving a response rate of 95.7%. Participants’ age ranged from 18 to more than 45 years, with a mean age of 34.6 years. Table 1 shows the socio-demographic characteristic of the study participants. Males comprised 51.8% (n=218), and females were 48.2% (n=203) of the study population. The majority of patients were married, 64.4% (n=217), and 26.8% (n=113) were single. Almost one-third, 35.6% (n=150), were in the age group (25-34) years.

**Table 1:** Sociodemographic characteristics of the respondents attending PHCCs in Jazan (n=421)

Characteristic	Frequency	Percent
Gender		
Male	218	51.8
Female	203	48.2
Residence		
Rural	195	37.8
Urban	262	62.2
Age group (Years)		
18-24	81	19.2
25-34	150	35.6
35-44	100	23.8
45+	85	20.2
Marital status		
Married	217	64.4
Single	113	26.8
Divorced	22	5.2
Windowed	15	3.6
Occupation		
Civil servant	174	41.3
Private sector employees	49	11.62
Entrepreneurial	30	7.1
Not working	140	33.3
Other	28	6.7
Level of Education		
illiterate	30	7.1
Primary	35	8.3
Intermediate	47	11.2
Secondary	127	30.2
University and above	182	43.2
Suffering from any chronic condition		
Yes	96	22.8
No	325	77.2
Total	420	100

Table 2 shows the patients’ satisfaction level with the new web-based medical appointment system “Mawid.” The overall level of satisfaction was very high at 94.3% with 95% C.I.: 91.7-96.1. A large proportion of study participants were highly satisfied with the new web-based medical appointment system “Mawid,” as nine statements scored 90% and above in level of satisfaction. About 89.3% of respondents were satisfied with the waiting period to meet the service, while almost only 3.8% were dissatisfied with the registration process. Those who were satisfied with the service generally accounted for 95.7% of the study participants. No significant difference was reported according to gender regarding the level of satisfaction except for satisfaction with the waiting time, where males (92.7%) were significantly more satisfied than females (85.7%) (*p* = 0.021).

**Table 2:** The level of patient's satisfaction with the new web-based medical appointment system “Mawid” (n=421)

Statements		All Participants		Gender				P-value
				Male		Female		
		N	%	N	%	N	%	
Satisfaction with the waiting time before obtaining the service	Satisfied	376	89.3%	202	92.7%	174	85.7%	0.021
	Not satisfied	45	10.7%	16	7.3%	29	14.3%	
Satisfaction with the services provided	Satisfied	403	95.7%	207	95.0%	196	96.6%	0.418
	Not satisfied	18	4.3%	11	5.0%	7	3.4%	
Satisfaction with the speed of the appointment	Satisfied	384	91.2%	203	93.1%	181	89.2%	0.152
	Not satisfied	37	8.8%	15	6.9%	22	10.8%	
The ease of access to the application and electronic services	Satisfied	395	93.8%	203	93.1%	192	94.6%	0.533
	Not satisfied	26	6.2%	15	6.9%	11	5.4%	
Clarity of the application and the system	Satisfied	393	93.3%	203	93.1%	190	93.6%	0.844
	Not satisfied	28	6.7%	15	6.9%	13	6.4%	
Easy to find the nearest PHCCs	Satisfied	403	95.7%	205	94.0%	198	97.5%	0.076
	Not satisfied	18	4.3%	13	6.0%	5	2.5%	
Are you satisfied with the appointments provided?	Satisfied	395	93.8%	201	92.2%	194	95.6%	0.152
	Not satisfied	26	6.2%	17	7.8%	9	4.4%	
PHCC commitment to the appointment provided	Satisfied	402	95.5%	208	95.4%	194	95.6%	0.940
	Not satisfied	19	4.5%	10	4.6%	9	4.4%	
Care of staff in the registration zone	Satisfied	400	95.0%	203	93.1%	197	97.0%	0.065
	Not satisfied	21	5.0%	15	6.9%	6	3.0%	
The registration process in the PHCC	Satisfied	405	96.2%	207	95.0%	198	97.5%	0.166
	Not satisfied	16	3.8%	11	5.0%	5	2.5%	
Overall level of Satisfaction = 94.3% with 95% CI [91.7-96.1]								

Respondent’s perception of the new scheduling system “Mawid” is presented in Table 3. The table shows 89.1% of the participants agreed that the appointment system regulates the number of patients, while 87.7% considered the appointment system reduces clinic crowding. More than half of respondents (61.8%) agreed that the community culture might limit the scheduling system’s use. The majority of participants, 87.6% and 88.2%, agreed that the web-based medical appointment systems “Mawid” gives the beneficiary access to the nearest PHCCs and allows them to book, manage, modify, and cancel their appointments effectively, respectively.

**Table 3:** Patient's perception of the new scheduling system “Mawid” (n=421)

Statements	Strongly agree, n (%)	Agree, n (%)	Neutral, n (%)	Disagree, n (%)	Strongly disagree, n (%)
The appointment booking system regulates the number of PHCC attendees	196(46.6)	179(42.5)	33(7.8)	13(3.1)	0
The appointment booking system is suitable for all Patient	154(36.6)	142(33.7)	74(17.6)	38(9)	13(3.1)
The appointment system reduces clinic Crowding	189 (44.9)	180 (42.8)	36 (8.6)	12 (2.9)	4 (1)
The appointment booking system has increased the availability of close dates	175(41.6)	177(42)	45(10.7)	15(3.6)	9(2.1)
There is an accumulation of the number of patients despite the use of the appointment booking system	64(15.2)	114(27.1)	78(18.5)	123(29.2)	42(10)
The economic aspect limits the use of the scheduling system (appointment)	53(12.6)	114(27.1)	109(25.9)	104(24.7)	41(9.7)
Community culture limits the use of the scheduling system (appointment)	117(27.8)	143(34.0)	65(15.4)	76(18.1)	20(4.8)
The system provides the beneficiary access to the nearest PHCCs	230(54.6)	139(33)	38(9)	10(2.4)	4(1)
The system allows the beneficiary to book, manage, modify and cancel appointments effectively	204(48.5)	167(39.7)	37(8.8)	10(2.4)	3(0.7)
The services provided through the system meet all my needs	188(44.7)	161(38.2)	50(11.9)	17(4)	5(1.2)

PaPatient'serception of the new scheduling system “Mawid” according to Gender is shown in Table 4. The table indicated that there are no differences in patients' perception according to gender as the p > 0.05 for all statements reflecting their perception.

**Table 4:** Patient's perception of the new scheduling system “Mawid” according to gender (n=421)

Statement	Mean	SD	Mean	SD	Mean	SD	P-value
The appointment booking system regulates the number of PHCC attendees	3.31	0.76	3.35	0.75	3.33	0.75	0.767
The appointment booking system is suitable for all Patient	2.91	1.12	2.93	1.05	2.92	1.08	0.446
The appointment system reduces clinic crowding	3.25	0.84	3.31	0.78	3.28	0.81	0.297
The appointment booking system has increased the availability of close dates	3.10	0.98	3.26	0.82	3.18	0.91	0.112
There is an accumulation of the number of patients despite the use of the appointment booking system	2.09	1.21	2.08	1.29	2.09	1.25	0.094
The economic aspect limits the use of the scheduling system (appointment)	2.12	1.23	2.04	1.14	2.09	1.19	0.207
Community culture limits the use of the scheduling system (appointment)	2.77	1.19	2.46	1.20	2.62	1.20	0.106
The system provides the beneficiary access to the nearest PHCCs	3.36	0.88	3.41	0.75	3.38	0.82	0.064
The system allows the beneficiary to book, manage, modify and cancel appointments	3.34	0.80	3.32	0.78	3.33	0.79	0.458
The services provided through the system meet all my needs	3.17	0.909	3.27	0.87	3.22	0.89	0.787

Univariate and multivariate logistic regression analysis suggested that male patients were more likely to have a higher level of satisfaction with the web-based appointment system as compared with female (COR = 2.95, 95% C.I.: 1.15-7.60, p=0.025) and (AOR = 3.12, 95% C.I.: 1.14-8.52, p=0.026), respectively. Also, patients in the age groups 25-34 and 35-44 years had a low level of satisfaction as compared to old patients (45 years and above) (COR = 0.31 and 0.23) and (AOR = 0.27 and 0.19) (p < 0.05 for all) (Table 5).

**Table 5:** Predictors of overall satisfaction based on univariate and multivariate logistic Regression analysis

Variable	COR	95% CI		P-value	AOR	95% CI		P-value
		Lower	Upper			Lower	Upper	
Gender								
Male	2.95	1.15	7.60	0.025	3.12	1.14	8.52	0.026
Female (Ref)	1				1			
Age groups (years)								
18-24	0.49	0.16	1.51	0.216	0.29	0.07	1.17	0.082
25-34	0.31	0.11	0.89	0.030	0.27	0.08	0.96	0.043
35-44	0.23	0.06	0.87	0.031	0.19	0.04	0.80	0.023
45+(Ref)	1				1			
Mode of Living								
Rural	1.42	0.62	3.26	0.403	1.10	0.46	2.65	0.833
Urban (Ref)	1							
Educational Level								
Educated	1.15	0.46	2.84	0.770	0.68	0.21	2.16	0.509
Well- educated (Ref)	1				1			
Work Status								
Working (Ref)	1							
Not working	1.47	0.63	3.39	0.370	0.51	0.20	1.32	0.166
Suffering from any Chronic condition								
Yes	1.14	0.44	2.95	0.792	0.58	0.18	1.89	0.364
No (Ref)	1				1			

## Discussion

There is increasing interest in the web-based appointment system, which can effectively increase patient satisfaction by reducing waiting times and non-attendance (no-show) rates [10,12]. A group of studies documented that adopting web-based appointment systems saves time and resources [6-12]. To the best of our knowledge, this study was the first to evaluate patient satisfaction with appointment scheduling systems at the PHCCs level in Jazan. The present study aimed to assess the patient’s perception and investigate the satisfaction level with the new web-based medical appointment system “Mawid.”

The overall level of satisfaction was very high in this study at 94.3%. This finding is higher than the results of the only available study that conducted in the Kingdom of Saudi Arabia [18] on the same topic, which produced an overall level of satisfaction of 48.1% with the “Mawid” application. The differences between the estimates may be attributed to the differences in the level of coverage in both studies, as Makkah’s study was conducted in one PHC Center. Our result is similar to a study conducted in Canada, where patients reported a high degree of usability and general satisfaction at 93% [20].

About 89.3% of respondents were satisfied with the waiting period to meet the service. In fact, most studies reported that appointment systems signifi­cantly reduce the time of arranging appointments and save the participants’ time [12]. This result is consistent with the results of studies [10,15,21-23], which have shown that appointment systems can effectively reduce waiting time.

Our study revealed a positive perception towards “Mawid” among patients, as 89.1% of the participants agreed that the appointment booking system regulates the number of patients, while 87.7% of participants considered that the appointment system reduces clinic crowding. The majority of participants, 87.6% and 88.2%, respectively, agreed that the web-based medical appointment systems “Mawid” gives the beneficiary access to the nearest PHCCs and allows them to book, manage, modify and cancel their appointments effectively. These results are consistent with many published research. In their review, Zhao et al. enumerated a number of benefits of adopting the web-based system, including reduced no-show rate, decreased staff labor, decreased waiting time, and improved satisfaction [12].

By analyzing the factors influencing the satisfaction level of web-based medical appointment systems “Mawid”, we found that satisfaction is significantly associated with gender. Many studies did not find a significant association between the level of satisfaction and gender [7,10,18,24]. We found that males were more satisfied than females with the service, this finding needs in-depth investigation, and a qualitative study may be suggested to explore these differences. Also, we found that young patients had lower satisfaction compared with older patients. A possible explanation is that older patients are more frequent users of the services than younger patients.

Our research has some limitations: first, the research was conducted in a number of selected PHCCs in the Jazan region, which decreases the potentiality of generalizing the study results to the other areas in KSA. Second, data were collected using a self-reported questionnaire, which is a potential error source. Third, patient satisfaction research should be handled with care as a patient’s expectations for his care encounter. Some research demonstrated that it is a subjective healthcare measure [25,26], as two patients receiving the same services may have different opinions. Despite these limitations, our study provided an estimate for the level of satisfaction with the “Mawid” appointment system for Jazan for the first time. Finally, this study was based on a cross-sectional study design. The associations mentioned in this study should be interpreted with care, as this design cannot determine cause and effect.

## Conclusions

In conclusion, this study revealed a high level of satisfaction among study participants with the new web-based medical appointment system “Mawid.” The system effectively improved patients’ perception and satisfaction with getting registration and reduced waiting times. The results of this study can provide a general picture of patients' views assessing the new web-based medical appointment System “Mawid,” because we conducted it following the implementation of the system. Patients' satisfaction can be assessed regularly and used systematically as a quality and benchmarking instrument in primary healthcare.
